# Study of Antiobesity Effect through Inhibition of Pancreatic Lipase Activity of* Diospyros kaki* Fruit and* Citrus unshiu* Peel

**DOI:** 10.1155/2016/1723042

**Published:** 2016-07-27

**Authors:** Gyo-Nam Kim, Mi-Rae Shin, Sung Ho Shin, Ah Reum Lee, Joo Young Lee, Bu-Il Seo, Min Yeong Kim, Tae Hoon Kim, Jeong Sook Noh, Man Hee Rhee, Seong-Soo Roh

**Affiliations:** ^1^Department of Food Science and Biotechnology, Kyungnam University, Gyeongsangnam-do 51767, Republic of Korea; ^2^College of Korean Medicine, Daegu Haany University, Gyeongsan 38610, Republic of Korea; ^3^Department of Food Science & Biotechnology, Daegu University, 201 Daegudae-ro, Gyeongsangbuk-do 712-714, Republic of Korea; ^4^Department of Food Science & Nutrition, Tongmyong University, Busan 48520, Republic of Korea; ^5^College of Veterinary Medicine, Kyungpook National University, Daegu 41566, Republic of Korea

## Abstract

Pancreatic lipase is the enzyme responsible for digestion and absorption of triglycerides, being its inhibition one of the widest studied methods used to determine the potential activity of natural products to inhibit dietary fat absorption. Decrease of energy intake from dietary fat through inhibition of this enzyme may be an excellent strategy to prevent and treat obesity. The inhibitory activity on pancreatic lipase enzyme of* Diospyros kaki* fruit and* Citrus unshiu* peel mixture extract (PCM) was evaluated* in vitro* and its antiobesity effects were studied based on the serum lipid parameters analysis from high-fat diet- (HFD-) fed mice* in vivo*. PCM was orally administered at a dose of 50 and 200 mg/kg body weight for 6 weeks. In addition, the activity of pancreatic lipase was assessed using orlistat (positive control). PCM exhibited inhibitory effect on lipase activity with IC_50_ value of 507.01 *μ*g/mL. Moreover, serum triacylglycerol, total cholesterol levels, and visceral fat weight were significantly reduced compared to HFD control mice in PCM 200 mg/kg-treated mice (*p* < 0.05). These results suggest that PCM administration may be a novel potential antiobesity agent for reduction of fat absorption via inhibition of pancreatic lipase.

## 1. Introduction

Obesity results from an energy imbalance and is now considered a serious and global health risk by the World Health Organization (WHO). It is associated with health problems like dyslipidemia, hypertension, fatty liver disease, diabetes mellitus, cancers, osteoarthritis, and asthma [1, 2]. In 2014, WHO reported that more than 1.9 billion adults, 18 years and above, stated as overweight and of these about more than 31% were obese [3]. Moreover, WHO predicted that this number will be elevated to approximately 3.3 billion by 2030 (about 1.7 times) [4]. According to this current trend, the economic burden of obesity (direct and indirect health care costs including health monitoring, nutritional supplements, and surgical management) is expected to grow every year. To date, orlistat (Xenical) approved by FDA, relatively effective drug for long-term treatment of obesity, exerts the drug efficacy through inhibition of pancreatic lipase enzyme and prevents the absorption of approximately 30% of dietary fat [5]. However, it is limited in its use due to severe gastrointestinal side effects. Herein, the prevention of the obesity may decrease the incidence of obesity-related diseases and lead to reducing immoderate costs and undesirable side effects. Hence, the recent obesity targets are focused on innoxious and therapeutic natural products. An inhibition of the dietary fat absorption is one of the common approaches in the effort to decrease an excessive energy intake [6].

Pancreatic lipase enzyme (triacylglycerol acylhydrolase) secreted from pancreas is a key enzyme related to the dietary triglycerides absorption and catalyzes the digestion of dietary triglycerides [7]. Among various lipases, pancreatic lipase performs the hydrolysis of 50–70% of total dietary fats [8]. The reduction of fat absorption through pancreatic lipase inhibition is known to benefit the regulation of obesity [9]. Hydrolysis activity of pancreatic lipase is maintained by Ser152, Asp176, and His263 amino acids; particularly, Ser152 is responsible for lipolysis activity [10].

PCM is herbal formulation of* Diospyros kaki* fruit (*Diospyros kaki* Thunb.) and* Citrus unshiu* peel (*Citrus unshiu* S. Marcov.). Both of these herbs are the most well-known traditional herbal medicines, frequently used to treat obesity [11–13]. Peel of* Citrus unshiu*, which is a seedless and easy-peeling citrus fruit, has been used for traditional herbal medicine in East-Asia including Korea.* Diospyros kaki* fruit contains a number of bioactive compounds, including polyphenols (especially tannins), carotenoids, flavonoids, vitamins, minerals, and dietary fiber [14, 15]. Particularly, tannins exert several biological effects, which involve antioxidant, anti-inflammatory, antitumor, antihypertensive, and antidiabetic activities [16, 17]. In addition,* Citrus unshiu* peel contains many phytochemicals such as hesperidin, naringin, and nobiletin [18]. The biological and multiple compounds of two herbs,* Diospyros kaki* fruit or* Citrus unshiu* peel, performed a pharmacological effect such as hypocholesterolemic [19], antiadipogenic [20], anti-inflammatory [21], antioxidant [22], antitumor [13] effects.

To the best of our knowledge, no study has previously investigated the antiobesity effect of* Diospyros kaki* fruit and* Citrus unshiu* peel mixture extract,* in vitro* or* in vivo*. Therefore, the present study aims to investigate the inhibition property on porcine pancreatic lipase* in vivo* and changes in body weight and serum lipid parameters and visceral fat weight on an experimental model of obesity.

## 2. Materials and Methods

### 2.1. Materials

Porcine pancreatic lipase (Type II), orlistat, morpholinepropanesulfonic acid (MOPS), Tris-HC1,* p*-nitrophenyl butyrate (*p*-NPB), 2,2-diphenyl-1-picrylhydrazyl (DPPH), and 2,2′-azino-bis-diammonium salt (ABTS) were purchased from Sigma-Aldrich Co. (St Louis, MO, USA). Ethylenediaminetetraacetic acid (EDTA) was purchased from Wako Pure Chemical Industries, Ltd. (Osaka, Japan). Viscozyme® was purchased from Novozymes (Denmark). All other reagents were of biochemical grade.

### 2.2. Preparation of the Mixture Extract

A unripe* Diospyros kaki* fruit (*Diospyros kaki* Thunb.) was harvested in Gyeongsangbuk-do Agricultural Research & Extension Services (Sangju, Korea) and a dried* Citrus unshiu* peel (*Citrus unshiu* S. Marcov.) was purchased from MSC Co., Ltd. (Yangsan, Korea). Each 500 kg was selected and extracted with 5 times of water and boiled in 100°C for 2 h. And then enzyme decomposition was carried out for 15 h. Next, enzyme (Viscozyme) was inactivated in 90°C for 30 min [23]. After filtration using the diatomite, the extracts were concentrated till 0 bx. The concentrated extracts added dextrin and were sterilized in 95°C for 30 min. The sterilized extracts were freeze-dried and powered by a grinder.

### 2.3. Experimental Animals and Treatment

Male healthy 4-week-old ICR mice (about 30–32 g) were purchased from Orient (Gyeonggi-do, Korea). Each mouse was kept at room temperature (22 ± 3°C) and humidity (55 ± 5%) with a 12-h light/dark cycle. The experiments were approved by the Ethics Committee of Animal Experimentation of the University of Daegu Haany. The mice were allowed free access to laboratory pellet chow and water ad libitum. After adaptation (1 week), all experimental mice except normal mice (*n* = 8) were fed with 60% high-fat diet (HFD; Diet 12492, Research Diets, Inc., New Brunswick, NJ, USA) for 5 days to adapt to a feed. Thereafter, ICR mice (*n* = 32) fed 60% HFD were randomly divided into four groups (*n* = 8 in each group): HFD control group, orlistat group (60 mg/kg/day), and two PCM treatment groups (50 and 200 mg/kg/day). The normal group is supplied with a normal feed and the rest of the groups are supplied with 60% HFD until the experimental end. The normal and HFD control groups were given water using a stomach tube, while the drug treatment groups were orally given orlistat or PCM daily using a stomach tube for 6 weeks. After administration for 6 weeks, each mouse was etherized after fasting for 12 h. The blood was immediately centrifuged at 1,500 ×g for 10 min at 4°C. Serum triglyceride and total cholesterol were conducted spectrophotometrically using commercially available kits (Wako Pure Chemical Industries, Ltd., Osaka, Japan). HDL-cholesterol is measured using a commercial kit from Asan Pharm Co., Ltd. (Hwaseong-si, Korea, Cat. AM203). LDL-cholesterol levels are calculated though TG, TC, and HDL levels:

(1) LDL-cholesterol  levelmg/dL=TC−HDL-cholesterol−TG5.

### 2.4. DPPH Radical Scavenging Activity of PCM

Antioxidant activity determination of PCM was performed by the DPPH radical scavenging according to the method of Park et al. [24]. 100 *μ*L of an ethanolic solution of PCM (blank: 100 *μ*L of ethanol) was added to 100 *μ*L of an ethanolic solution of DPPH (60 *μ*M) using 96-well plate. The ascorbic acid (standard sample) was prepared for eight concentrations (0.5, 1, 2, 5, 10, 20, 50, and 100 *μ*g/mL). The PCM was prepared for six concentrations (5, 10, 20, 50, 100, and 200 *μ*g/mL). The reaction mixture was incubated in the dark at 25°C for 30 min. The optical density was determined using a microplate reader model infinite M200 PRO (Tecan, Austria). The mixture was measured spectrophotometrically at 540 nm. The antioxidant activity of each sample was expressed in terms of IC_50_ (micromolar concentration required to inhibit DPPH radical formation by 50%, calculated from the log-dose inhibition curve). The radical scavenging activity was calculated as a percentage using the following equation:

(2) DPPH  radical  scavenging  activity%=1−AsampleAblank×100.

### 2.5. ABTS Radical Scavenging Activity of PCM

ABTS radical scavenging activity of the different extracts was measured according to the modified method of Re et al. [25]. ABTS stock solution was dissolved in water to 7.4 mM concentration. The ABTS radical cation (ABTS) was produced by reacting ABTS stock solution with 2.45 mM potassium persulfate and allowing the mixture to stand for 14 h at room temperature in the dark. The ABTS solution was diluted with ethanol to obtain an absorbance of 0.70 ± 0.02 at 750 nm. After adding 95 *μ*L of diluted ABTS solution (*A*_750 nm_ = 0.70 ± 0.02) to 5 *μ*L of sample, the mixture was left at room temperature for 15 min in the dark. The absorbance at 750 nm was measured using a microplate reader model infinite M200 PRO (Tecan, Austria). The blank was prepared in the same manner, except distilled water was used instead of the sample. The radical scavenging activity was calculated as a percentage using the following equation:

(3) ABTS  radical  scavenging  activity%=1−AsampleAblank×100.

### 2.6. Measurement of Porcine Pancreatic Lipase Inhibitory

Pancreatic lipase activity was modified from the method previously reported by Kim et al. [26]. Briefly, an enzyme buffer was prepared by the addition of 6 *μ*L of a solution of porcine pancreatic lipase (Sigma-Aldrich) in buffer containing 10 mM MOPS (morpholinepropanesulfonic acid) and 1 mM EDTA, pH 6.8, to 169 *μ*L Tris buffer (100 mM Tris-HC1 and 5 mM CaCl_2_, pH 7.0). Then, either 20 *μ*L of PCM at the test concentration (100, 250, 500, and 1000 *μ*g/mL) or orlistat (0.1, 0.25, 0.5, and 1 *μ*g/mL) was mixed with 175 *μ*L enzyme buffer and incubated for 15 min at 37°C with 5 *μ*L of the substrate solution [10 mM* p*-NPB (*p*-nitrophenyl butyrate) in dimethylformamide]. The enzymatic reactions were allowed to proceed for 35 min at 37°C. Lipase activity was determined by measuring the hydrolysis of* p*-NPB into* p*-nitrophenol. Increase in light absorption at 405 nm was measured using a microplate reader, model infinite M200 PRO (Tecan, Austria). Inhibition of lipase activity was expressed as the percentage decrease in OD when porcine pancreatic lipase was incubated with the test compounds. Lipase inhibition (%) was calculated according the following formula:

(4) $$\begin{array}{c} \text{Inhibition}\left(\;\%\;\right)=100-\left[\;\frac{\left(B-b\right)}{\left(A-a\right)}*100\;\right], \end{array}$$

where *A* is the activity without inhibitor, *a* is the negative control without inhibitor, *B* is the activity with inhibitor, and *b* is the negative control with inhibitor. The results were expressed as an average (*n* = 4).

### 2.7. Total Phenolic and Flavonoid Contents

The total phenolic content of PCM was quantified by mild modification from the method of Folin-Ciocalteu [27]. 10 *μ*L PCM and distilled water 790 *μ*L were shaken well and then mixed with 50 *μ*L of Folin-Ciocalteu's reagent for 1 min. After that 150 *μ*L of 20% sodium carbonate solution (Na_2_CO_3_) was added and the mixture was shaken for 2 h at 20°C. Finally, the absorbance of the resulting color was measured at 765 nm. The total phenolic content was expressed as mg gallic acid equivalents per gram extract. Values presented are the average of three measurements. Flavonoid was extracted and quantified by adaptation of the method of Lister et al. [28]. PCM 50 *μ*L and 500 *μ*L diethylene glycol were mixed well. And then 1 N NaOH 5 *μ*L was added and the mixture was incubated for 1 h at 37°C. Finally, the absorbance of the resulting color was measured at 420 nm. The flavonoid content was expressed as mg naringin equivalents per gram extract. Values presented are the average of three measurements.

### 2.8. Statistical Analysis

Data are expressed as mean ± SEM. Statistical comparisons were assessed by one-way ANOVA followed by Dunnett's multiple comparison test (SPSS 18.0 for Windows, SPSS Inc., USA) and values of *p* < 0.05 were considered significant. Also, simple regression analysis was performed to investigate the correlation between DPPH radical scavenging and ABTS radical scavenging using the Microsoft Excel 2010 statistical package.

## 3. Results and Discussion

**Figure 1:**
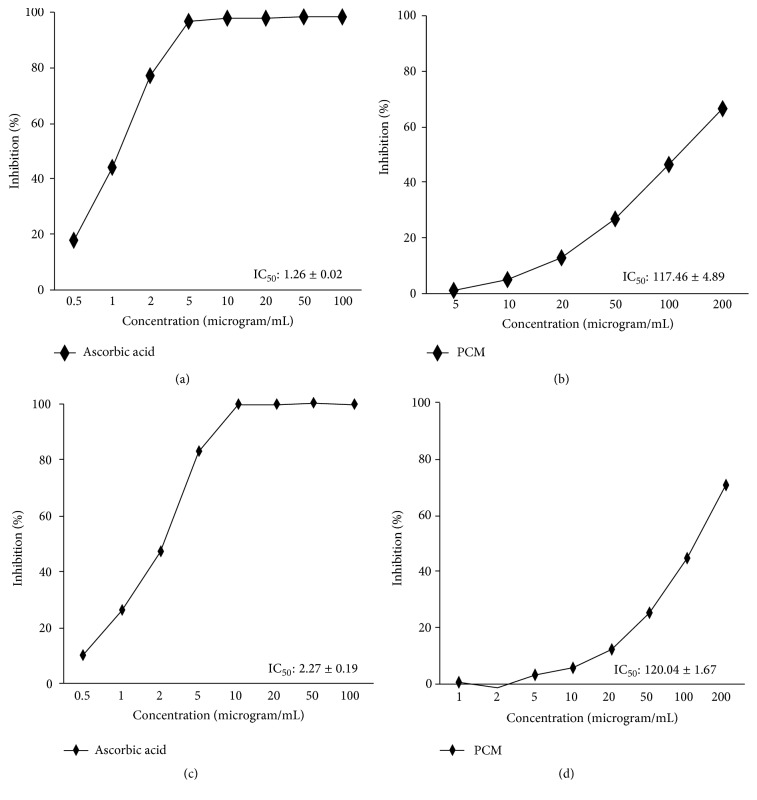
DPPH radical scavenging activity (a, b) and ABTS radical scavenging activity (c, d). (a, c) Ascorbic acid and (b, d) PCM,* Diospyros kaki* fruit and* Citrus unshiu* peel mixture extract. Each experiment was run in triplicate. The ascorbic acid was used as standard sample.

Antioxidant agents from natural herbs have received a potent attention since they are safe and lesser toxic [29]. This study was performed to determine the antioxidant activity of PCM through DPPH and ABTS radical scavenging activity [30]. Antioxidant activity is expressed in terms of IC_50,_ and a lower IC_50_ value corresponds to a larger scavenging activity. IC_50_ (*μ*g/mL) represents half maximal concentration of tested compounds to scavenge DPPH and ABTS radical. As shown in Figures 1(a) and 1(b), IC_50_ of DPPH radical scavenging activity of PCM was found to be 117.46 ± 4.89 *μ*g/mL and IC_50_ value of ascorbic acid (positive control) as a positive control was 1.26 ± 0.02 *μ*g/mL. The calculated IC_50_ value of PCM against the ABTS radical was determined to be 120.04 ± 1.67 *μ*g/mL and IC_50_ value of ascorbic acid as a positive control was 2.27 ± 0.19 *μ*g/mL (Figures 1(c) and 1(d)).

Moreover, the phenolic compound or flavonoid performs as another antioxidant agent by chelating redox-active metal ions, inactivating lipid free radical chains, and avoiding the hydroperoxide conversions into reactive oxyradicals [30, 31]. Total phenolic content was measured as gallic acid equivalents (GAE) with reference to standard curve (*y* = 0.023*x* + 0.30 and *R* ^2^ = 0.997) and was found to be 29.90 ± 0.14 mg GAE/g of PCM extract. The flavonoid content was 18.33 ± 0.08 mg naringin equivalent (NE)/g of PCM extract, respectively, with reference to standard curve (*y* = 0.0195*x* + 0.04 and *R* ^2^ = 0.9999). The total phenolic content was expressed as gallic acid equivalents and flavonoid content was expressed as rutin or naringin equivalents varied according to extract condition or developmental stage. However, young persimmon used in this experiment is higher than old persimmon used by a different researcher in two contents based on our current study [32, 33].

**Figure 2:**
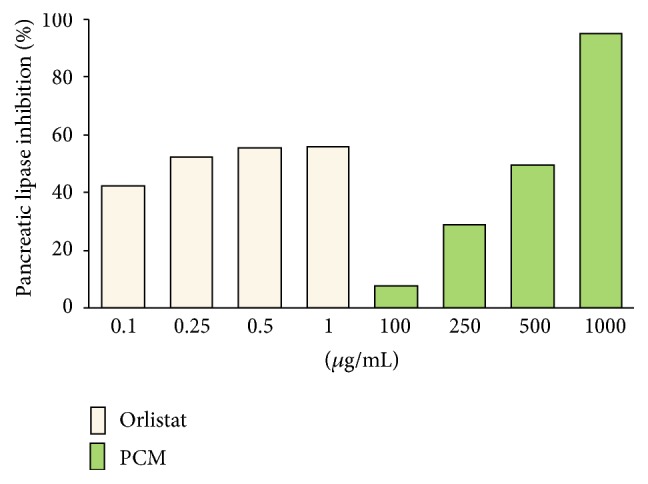
Inhibition of pancreatic lipase by PCM. Orlistat was used as positive control. PCM,* Diospyros kaki* fruit and* Citrus unshiu* peel mixture extract.

Dietary fats are mainly comprised of mixed triglycerides about 90% and need to be hydrolyzed by the various lipases for their absorption. The main human lipases to digest dietary fats include lingual, gastric, and pancreatic lipases [34]. Among these lipases, pancreatic lipase is responsible for the hydrolysis of 50–70% of dietary fats to fatty acids (FA) and monoglycerides (MG). These are released by lipid hydrolysis and form mixed micelles with bile salts, cholesterol, and lysophosphatidic acid. And then mixed micelles are absorbed into enterocytes where resynthesis of TG happens. Finally, triglyceride is stored in adipocytes as the major source of energy [8]. Recently, new approaches for the treatment of obesity tried to reduce energy intake through gastrointestinal mechanisms, without altering any vital mechanisms. Pancreatic lipase inhibition is one of such attempts and many researchers focused on the potential efficacy of natural products as antiobesity agents [35]. In the* in vitro* experiment, the ability of extract had condensed tannins as an inhibitor of pancreatic lipase seems to be well demonstrated [36]. In our current study, the obtained result showed that PCM inhibited pancreatic lipase activity with IC_50_ of 507.01 *μ*g/mL compared to orlistat with IC_50_ of 0.218 *μ*g/mL (Figure 2). PCM was not more effective than positive control (orlistat). However, the pancreatic lipase inhibitory effect of PCM was superior to those when various dietary plants were screened by Conforti et al. [37].

**Figure 3:**
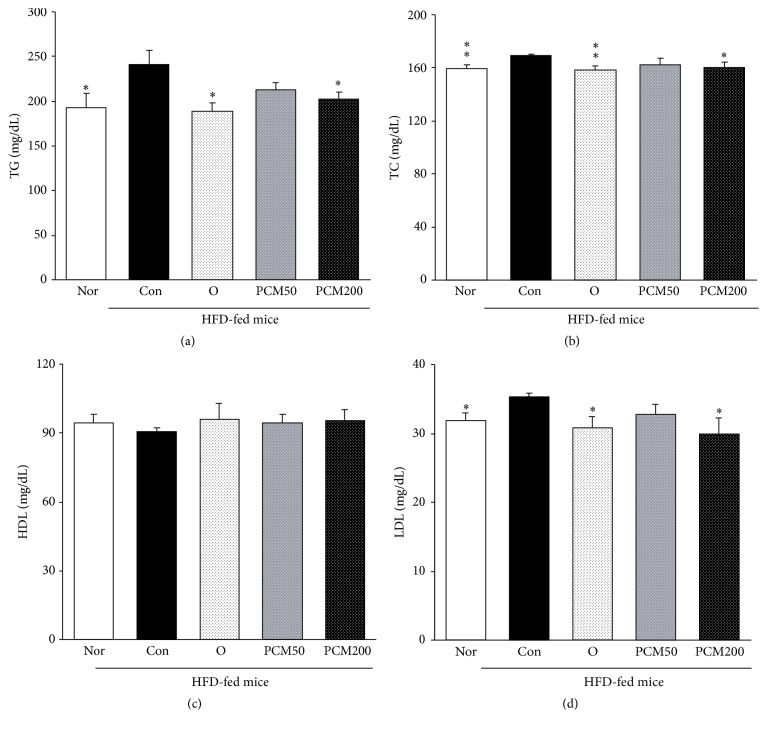
Serum triglyceride, total cholesterol, HDL-cholesterol, and LDL-cholesterol levels. Con, HFD control mice; O, orlistat 60 mg/kg-treated and HFD-fed mice; PCM50, PCM 50 mg/kg-treated and HFD-fed mice. PCM200, PCM 200 mg/kg-treated and HFD-fed mice. Data are the mean ± SEM, *n* = 8. Significance: ^*∗*^ *p* < 0.05 and ^*∗∗*^ *p* < 0.01 versus HFD control mice.

Ultimately, obesity causes abdominal visceral fat and weight change due to HFD. An excessive accumulation of fat in the abdominal viscera is associated with the increase of serum TG, TC, and LDL, which are related to hyperlipidemia [38]. To evaluate the effects of PCM on HFD-induced obesity, we investigated the development of HFD-induced obesity in mice with and without PCM supplementation for 6 weeks and measured the effects of PCM on serum lipid profiles such as TG, TC, HDL-cholesterol, and LDL-cholesterol at the experimental end.  Table 1 shows the body weight change and visceral fat weight during the experimental periods. As shown in Table 1, HFD control mice increased significantly final body weight compared with normal mice (55.15 ± 1.78 g, 47.92 ± 1.46 g, resp., *p* < 0.01). In addition, the visceral fat weight in HFD control mice was significantly increased compared to normal mice (238% of normal value), but orlistat and PCM200-treated mice were significantly decreased compared with those of HFD control mice. PCM50 treatment showed a tendency to decrease (without significance). Above all, body weight change reduced significantly in all drug-treated experimental groups. Overall, PCM may help to improve the disorders of HFD-induced obesity. Besides, Figure 3 showed that high-fat diets caused a marked increase in serum TG, TC, and LDL-cholesterol (*p* < 0.05, *p* < 0.01, and *p* < 0.05, resp., except HDL-cholesterol). The augmented TG, TC, and LDL-cholesterol levels were significantly lowered by orlistat and PCM200 treatment compared with the HFD control mice, whereas the administration of orlistat and PCM slightly elevated the HDL level (without significance).

## 4. Conclusion

The present study conducted how PCM exerts antiobesity effect related to antioxidant and pancreatic lipase activities. Taken together, PCM inhibited triglyceride absorption via the inhibition of pancreatic lipase and had preventive effects on suppressing serum lipid parameters and visceral fat accumulation in HFD-fed obese mice. These results suggest that PCM administration may attenuate some of the physiological changes that occur in obesity.

## Figures and Tables

**Table 1:** The effect of PCM on body weight and visceral fat weight.

Group	Body weight			Visceral fat weight
	Initial (g)	Final (g)	Change (g/6 weeks)	(g)
Normal	36.06 ± 0.96	47.92 ± 1.46^*∗∗*^	11.86 ± 1.00^*∗*^	24.1 ± 1.0^*∗∗∗*^
HFD-fed mice				
Con	37.67 ± 0.51	55.15 ± 1.78	17.48 ± 1.95	57.5 ± 1.5
O	36.71 ± 0.96	44.92 ± 1.62^*∗∗*^	8.21 ± 1.82^*∗∗*^	42.5 ± 2.8^*∗*^
PCM50	37.18 ± 0.44	48.06 ± 0.17^*∗∗*^	10.88 ± 0.52^*∗*^	50.6 ± 3.2
PCM200	36.93 ± 0.38	45.98 ± 0.94^*∗∗*^	9.05 ± 0.90^*∗∗*^	50.3 ± 2.3^*∗*^

Con, HFD control mice; O, orlistat 60 mg/kg-treated and HFD-fed mice; PCM50, PCM 50 mg/kg-treated and HFD-fed mice.

PCM 200, PCM 200 mg/kg-treated and HFD-fed mice. Data are the mean ± SEM, *n* = 8.

Significance: ^*∗*^ *p* < 0.05, ^*∗∗*^ *p* < 0.01, and ^*∗∗∗*^ *p* < 0.001 versus HFD control mice.

## References

[1] Billington C. J., Epstein L. H., Goodwin N. J. (2000). Overweight, obesity, and health risk. *Archives of Internal Medicine*.

[2] Kopelman P. G. (2000). Obesity as a medical problem. *Nature*.

[3] http://www.who.int/mediacentre/factsheets/fs311/en/.

[4] Wei K., Wang G.-Q., Bai X. (2015). Structure-based optimization and biological evaluation of pancreatic lipase inhibitors as novel potential antiobesity agents. *Natural Products and Bioprospecting*.

[5] Seyedan A., Alshawsh M. A., Alshagga M. A., Koosha S., Mohamed Z. (2015). Medicinal plants and their inhibitory activities against pancreatic lipase: a review. *Evidence-Based Complementary and Alternative Medicine*.

[6] De La Garza A. L., Milagro F. I., Boque N., Campión J., Martínez J. A. (2011). Natural inhibitors of pancreatic lipase as new players in obesity treatment. *Planta Medica*.

[7] Veeramachaneni G. K., Raj K. K., Chalasani L. M., Annamraju S. K., Js B., Talluri V. (2015). Shape based virtual screening and molecular docking towards designing novel pancreatic lipase inhibitors. *Bioinformation*.

[8] Birari R. B., Bhutani K. K. (2007). Pancreatic lipase inhibitors from natural sources: unexplored potential. *Drug Discovery Today*.

[9] Ahn J. H., Liu Q., Lee C. (2012). A new pancreatic lipase inhibitor from *Broussonetia kanzinoki*. *Bioorganic and Medicinal Chemistry Letters*.

[10] Bourne Y., Martinez C., Kerfelec B., Lombardo D., Chapus C., Cambillau C. (1994). Horse pancreatic lipase: the crystal structure refined at 2.3 Å resolution. *Journal of Molecular Biology*.

[11] Matsumoto K., Yokoyama S.-I., Gato N. (2008). Hypolipidemic effect of young persimmon fruit in C57BL/6.KOR-*ApoE*
^shl^ mice. *Bioscience, Biotechnology and Biochemistry*.

[12] Lim D. W., Lee Y., Kim Y. T. (2014). Preventive effects of *Citrus unshiu* peel extracts on bone and lipid metabolism in OVX rats. *Molecules*.

[13] Lee S., Ra J., Song J.-Y. (2011). Extracts from Citrus unshiu promote immune-mediated inhibition of tumor growth in a murine renal cell carcinoma model. *Journal of Ethnopharmacology*.

[14] Kim H.-H., Kim D.-S., Kim S.-W. (2013). Inhibitory effects of Diospyros kaki in a model of allergic inflammation: role of cAMP, calcium and nuclear factor-*κ*B. *International Journal of Molecular Medicine*.

[15] Matsumoto K., Kadowaki A., Ozaki N. (2011). Bile acid-binding ability of Kaki-tannin from young fruits of persimmon (*Diospyros kaki*) in vitro and in vivo. *Phytotherapy Research*.

[16] Matsumura Y., Ito T., Yano H. (2016). Antioxidant potential in non-extractable fractions of dried persimmon (*Diospyros kaki* Thunb.). *Food Chemistry*.

[17] Forouzanfar F., Torabi S., Askari V. R., Asadpour E., Sadeghnia H. R. (2016). Protective effect of diospyros kaki against glucose-oxygen-serum deprivation-induced PC12 cells injury. *Advances in Pharmacological Sciences*.

[18] Lu Y., Zhang C., Bucheli P., Wei D. (2006). Citrus flavonoids in fruit and traditional chinese medicinal food ingredients in China. *Plant Foods for Human Nutrition*.

[19] Gato N., Kadowaki A., Hashimoto N., Yokoyama S.-I., Matsumoto K. (2013). Persimmon fruit tannin-rich fiber reduces cholesterol levels in humans. *Annals of Nutrition and Metabolism*.

[20] Lim H., Yeo E., Song E. (2015). Bioconversion of Citrus unshiu peel extracts with cytolase suppresses adipogenic activity in 3T3-l1 cells. *Nutrition Research and Practice*.

[21] Oh Y.-C., Cho W.-K., Jeong Y. H. (2012). Anti-inflammatory effect of *Citrus unshiu* peel in LPS-stimulated RAW 264.7 macrophage cells. *The American Journal of Chinese Medicine*.

[22] Jang I.-C., Jo E.-K., Bae M.-S. (2010). Antioxidant and antigenotoxic activities of different parts of persimmon (Diospyros kaki cv. Fuyu) fruit. *Journal of Medicinal Plants Research*.

[23] Im H., Yoon K. (2016). Production and characterisation of alcohol-insoluble dietary fibre as a potential source for functional carbohydrates produced by enzymatic depolymerisation of buckwheat hulls. *Czech Journal of Food Sciences*.

[24] Park C. H., Tanaka T., Kim H. Y., Park J. C., Yokozawa T. (2012). Protective effects of corni fructus against advanced glycation endproducts and radical scavenging. *Evidence-Based Complementary and Alternative Medicine*.

[25] Re R., Pellegrini N., Proteggente A., Pannala A., Yang M., Rice-Evans C. (1999). Antioxidant activity applying an improved ABTS radical cation decolorization assay. *Free Radical Biology and Medicine*.

[26] Kim J. H., Kim H. J., Park H. W., Youn S. H., Choi D.-Y., Shin C. S. (2007). Development of inhibitors against lipase and *α*-glucosidase from derivatives of monascus pigment. *FEMS Microbiology Letters*.

[27] Slinkard K., Singleton V. L. (1997). Total phenol analysis: automation and comparison with manual methods. *American Journal of Enology and Viticulture*.

[28] Lister C. E., Lancaster J. E., Walker J. R. L. (1996). Developmental changes in the concentration and composition of flavonoids in skin of a red and a green apple cultivar. *Journal of the Science of Food and Agriculture*.

[29] Ratnam D. V., Ankola D. D., Bhardwaj V., Sahana D. K., Kumar M. N. V. R. (2006). Role of antioxidants in prophylaxis and therapy: a pharmaceutical perspective. *Journal of Controlled Release*.

[30] Patro G., Bhattamisra S. K., Mohanty B. K., Sahoo H. B. (2016). *In vitro* and *in vivo* antioxidant evaluation and estimation of total phenolic, flavonoidal content of *Mimosa pudica* L. *Pharmacognosy Research*.

[31] Yang X., Kang S.-M., Jeon B.-T. (2011). Isolation and identification of an antioxidant flavonoid compound from citrus-processing by-product. *Journal of the Science of Food and Agriculture*.

[32] Lee J. H., Lee Y. B., Seo W. D., Kang S. T., Lim J. W., Cho K. M. (2012). Comparative studies of antioxidant activities and nutritional constituents of persimmon juice (Diospyros kaki L. cv. Gapjubaekmok). *Preventive Nutrition and Food Science*.

[33] Ercisli S., Akbulut M., Ozdemir O., Sengul M., Orhan E. (2008). Phenolic and antioxidant diversity among persimmon (*Diospyrus kaki* L.) genotypes in Turkey. *International Journal of Food Sciences and Nutrition*.

[34] Mukherjee M. (2003). Human digestive and metabolic lipases—a brief review. *Journal of Molecular Catalysis B: Enzymatic*.

[35] Yun J. W. (2010). Possible anti-obesity therapeutics from nature-a review. *Phytochemistry*.

[36] Oliveira R. F., Gonçalves G. A., Inácio F. D. (2015). Inhibition of pancreatic lipase and triacylglycerol intestinal absorption by a *Pinhão* coat (*Araucaria angustifolia*) extract rich in condensed tannin. *Nutrients*.

[37] Conforti F., Perri V., Menichini F. (2012). Wild mediterranean dietary plants as inhibitors of pancreatic lipase. *Phytotherapy Research*.

[38] Lee J. S., Kim K.-J., Kim Y.-H. (2014). Codonopsis lanceolata extract prevents diet-Induced obesity in C57BL/6 mice. *Nutrients*.

